# NAVIGATING AND ENHANCING HEALTHCARE COMMUNICATION: CHALLENGES AND STRATEGIES FOR LATE-DEAFENED OLDER ADULTS

**DOI:** 10.1093/geroni/igae098.4268

**Published:** 2024-12-31

**Authors:** Gianna Kohl, Erin Heniff, Maya Dye, Wendy Rogers

**Affiliations:** University of Illinois Urbana-Champaign, Champaign, Illinois, United States; University of Illinois Urbana-Champaign, Champaign, Illinois, United States; University of Illinois Urbana-Champaign, Champaign, Illinois, United States; University of Illinois Urbana-Champaign, Champaign, Illinois, United States

## Abstract

Effective communication in healthcare is crucial, especially for older adults with complex health issues. Late-deafened older adults, who develop severe to profound bilateral hearing loss post-lingually, face unique challenges in communicating their health needs. This issue is not well understood, and research describing strategies to manage these communication challenges in clinical settings is lacking. Understanding these challenges and responses for healthcare activities is essential to support late-deafened older adults and health professionals, ensuring quality care. Data were collected through the Aging Concerns, Challenges, and Everyday Solutions (ACCESS) study wherein questionnaires and interviews were completed by 56 late-deafened participants aged 60 to 80 (M = 69.4, SD = 6.0), living with a diagnosis of hearing loss for at least ten years (age of onset: M = 31.8, SD = 19.9). We conducted a detailed content analysis of interview data about challenges and response strategies in healthcare settings. The most difficult activity was participating in healthcare appointments (n = 38), followed by managing health, diet, and nutrition (n = 13). Communication barriers included conversational partners using verbal communication and insufficient visual cues, lack of support, and unclear speech. Response strategies included using external assistive technology, adaptive communication strategies (e.g., lip-reading), and educating healthcare providers about their needs. Our findings highlight the need for health professionals in clinical practice to understand and adapt to the specific communication challenges faced by late-deafened older adults. Implementing effective communication strategies and providing tailored support can lead to more accurate diagnosis and treatment, ultimately enhancing patient care.

